# The contribution of non-primary caregivers in early stimulation in Kenya and Uganda: Implications for ECD parenting programs in low- and middle-income countries

**DOI:** 10.1371/journal.pone.0323830

**Published:** 2025-05-23

**Authors:** Eunsoo Timothy Kim, Darcy Strouse, Sandra Sandoval, Lukiya Kibone, Damaris Wambua, Michela Profeta

**Affiliations:** 1 Program Resources and Institutional Learning, ChildFund International, Richmond, Virginia, United States of America; 2 ChildFund Uganda, Kampala, Uganda; 3 ChildFund Kenya, Nairobi, Kenya; Jawaharlal Institute of Postgraduate Medical Education and Research, INDIA

## Abstract

Parenting programs have primarily focused on supporting mothers with knowledge and practice of responsive caregiving. However, the role of non-primary caregivers such as grandparents, aunts, and older siblings has not been adequately addressed in programming. Using ChildFund International’s programmatic data from Kenya and Uganda, this cross-sectional study examined the extent to which children aged 0–5 years were left in the care of non-primary caregivers in the household and whether the primary caregivers’ absence was associated with engagement by non-primary caregivers. We found that a considerable proportion of children aged 0–5 years in Kenya and Uganda were entrusted in the care of non-primary caregivers for at least 3 days or more during the week. Yet, the primary caregivers’ absence was not consistently associated with greater engagement by fathers and other members of the household. Our findings call for parenting programs to consider adopting a holistic and contextualized approach where all involved caregivers are intentionally targeted rather than focusing just on the identified primary caregiver alone.

## Introduction

The 2030 Sustainable Development Goals issued a call for greater emphasis on responsive caregiving, early learning and early child development [1]. Target 4.2 states that by 2030 the goal is to “ensure that all girls and boys have access to quality early childhood development, care and pre-primary education so that they are ready for primary education [1].” It is well known that early child development is associated with a host of learning and behavioral outcomes in school and consequently, income earning potential as adults [2]. Early child development is therefore a very important factor that can change the trajectory of the child’s life course and subsequently, even outcomes of their children [2].There is already a significant evidence base that improved quality of maternal interaction with children increases the likelihood of their meeting critical developmental milestones [3]. Many programs have thus focused on improving the mother-child relationship and mother’s knowledge and practices of responsive caregiving related to feeding and stimulation in low- and middle-income countries (LMICs) (4–39). A 2021 meta-analysis study of parenting interventions with a randomized controlled design found that participation in the interventions resulted in less behavioral problems and better attachment as well as improved cognitive, language, motor, and socioemotional development for children [4,5,16,27,34–39]. For parents, their parenting knowledge, practices, and interactions with children significantly improved [6–15,17–26,28–33]. Importantly, the pooled effects were stronger for LMICs and most of the interventions implemented in LMICs primarily targeted the maternal primary caregivers [3].

There has also been increasing discourse and recognition on the role of paternal stimulation and responsive caregiving in early child development [40–46]. A study examining paternal stimulation in 38 countries found that children of highly engaged fathers had small but statistically significant increases in developmental scores compared to children of fathers with moderate or low stimulation [40]. Such associations were found to be even more pronounced when the engagement of mothers was low. These findings highlight the importance of including male caregivers in relevant programs and imply that paternal stimulation may be protective in circumstances where mothers are not able to attend to their children [40].

As evidenced by prior studies, focusing programmatic efforts on maternal and paternal engagement with children is well justified. Yet, parents are not always available in the home to care for their children [47,48]. Even if they are present, they may not always engage in responsive early stimulation with their children, or the quality of their engagement may not be at desired levels [47,48]. During the parents’ absence, young children are left in the care of another adult (e.g., grandmothers, aunts, or other adult females) or older siblings [47,48] and sometimes even placed under inadequate supervision (i.e., left alone or with another child under 10 years old) in the household [49–53].

In addition, kinship care and child fostering have traditionally been common in Africa, even for children with living biological caregivers [54]. A recent narrative review of these care practices in the literature as well as case studies from Africa found that the proportion of mothers sending at least one child under 16 years of age to kinship care or fostering ranged from 16.4% in Kenya to 45.72% in Namibia [54]. Reasons for these arrangements were not only motivated by the loss of biological caregivers or crisis situations, but they were also related to seeking educational and work opportunities, providing labor, strengthening family and community ties, and longstanding cultural beliefs regarding the general benefits of kinship care and child fostering [54]. While there could be benefits to this type of arrangement, there was also evidence that the fostered child living in a household with the caregivers’ biological children potentially faced differential treatment and disadvantages for opportunities [54]. Furthermore, little is known about the length of kinship care and fostering and the extent to which children shift in and out of these arrangements [54], especially when those offering kinship care live in proximity.

Given the frequent occurrence of kinship care, fostering, and non-primary caregivers in the same household sharing the responsibility of caregiving, focusing primarily on biological and/or step mothers and fathers in interventions may overlook the critical needs of young children in various living arrangements and circumstances. Yet, there has been very little interest in examining the contribution of other family members and relatives in early stimulation. Only a handful of studies have considered or discussed their level of engagement in early stimulation [16,42,54–56] and very few parenting programs in LMICs have intentionally included non-primary caregivers and considered the magnitude of their roles in children’s developmental outcomes [16,54,57,58].

While recognizing the complexity and the fluidity of caregiving arrangements for children in Africa, the current study aims to generate useful insights about the practice of caregiving in and across households where primary and non-primary caregivers both may share the role of caregiving. Findings are also expected to raise awareness of the need for programmatically including all potential caregivers of the child rather than limiting the programs’ target to just the ones identified as primary caregivers. For the context of Kenya and Uganda, we will specifically examine: [1] the extent to which children aged 0–5 years are left in the care of another adult in the household; and [2] whether the primary caregivers’ absence is associated with engagement by the mother, father, and other household members.

## Methods

### Study design and sample

The study conducted cross-sectional analysis with the baseline programmatic data collected for ChildFund International’s (ChildFund) Responsive and Protective Parenting (RPP) Program Model in Kenya and Uganda [59]. The main objective of the RPP Program Model was to “ensure that from pregnancy through age 5, children enjoy improved development and early learning outcomes and are protected at home and in their communities” [59]. The RPP Program Model was implemented over a period of 18 months and included community-led information sessions, capacity building workshops, and monthly group and home parenting sessions [59]. In Kenya, baseline programmatic data for RPP were collected in January and February of 2021 in the areas of Homa Bay, Kitui, Laikipia, Machakos, and Nyeri. In Uganda, baseline programmatic data were collected in May of 2022 in the areas of Masindi, Gulu, and Soroti. Across these settings, data contained responses from eligible primary caregivers who were willing to participate in the RPP Program. These initially included both female and male, and biological and non-biological parents, grandparents and relatives who spend the most time with the child under 5 years old and provide for most of their needs. In Kenya, the total sample was 1,526 respondents and in Uganda, the total sample was 838 respondents. However, the final analysis samples for this study in Kenya and Uganda only included the biological, maternal primary caregivers with a child 0–5 years old. This is because the majority of the caregiver survey respondents were biological, maternal primary caregivers. A total of 1281 women and 546 women in Kenya and Uganda were thus included in the final analysis samples respectively.

### Study setting

According to the 2022 Kenya Demographic and Health Survey, the average household size was 3.7 members, with a third of the households being led by women [60]. The total fertility rate for women in Kenya was about 3.4 children in 2022 [60]. The presence of one or more co-wives was reported by 9% of married women aged 15–49 years [60]. For children aged 5 at the beginning of the school year, 66% attended early childhood education and 22% attended primary school [60]. Seventy-eight percent of children aged 24–59 months in Kenya were developmentally on track according to the Early Childhood Development Index [60]. As for employment, 67% of married women aged 15–49 years were employed in the last 12 months [60]. Around 80% of married women aged 15–49 years made decisions about their own health care, participated in decisions about major household purchases and participated in decisions about visiting family or relatives [60].

According to the 2022 Uganda Demographic and Health Survey, the average household size was 4.5 members, with a third of the households being led by women [61]. The total fertility rate for women in Uganda was 5.2 children in 2022 [61]. Among married women aged 15–49 years in Uganda, 23% reported the presence of one or more co-wives [61]. Attendance to early childhood education programs was 29% for children aged 36–59 months [61]. Fifty-six percent of children aged 24–59 months in Uganda were developmentally on track according to the Early Childhood Development Index [61]. Among women aged 15–49 years, about 63% were employed in the last 12 months while 60% were currently employed at the time of the survey [61]. Seventy-eight percent of married women aged 15–49 years made decisions about their own health care, 74% participated in decisions around making major household purchases, and 80% participated in decisions about visiting family or relatives [61].

### Main outcomes and predictors

Survey respondents (i.e., biological, maternal primary caregivers) were asked about 6 early stimulation activities that they practiced themselves and the activities that they believed were practiced by the father and other household members in the past 3 days. These early stimulation activities are also listed in the UNICEF MICS surveys [62] and include reading books or looking at picture books with the child, telling stories to the child, singing songs to or with the child (including lullabies), taking the child outside the home, playing with the child and naming, counting or drawing things for or with the child. Based on these 6 early stimulation activities, UNICEF assesses whether four or more activities have been performed with children as an indicator of children receiving satisfactory dosage of early stimulation. The main outcomes of the current study were therefore engagement in 4 or more early stimulation activities by the mother in the past 3 days, engagement in 4 or more early stimulation activities by the father in the past 3 days (according to the mother’s report) and engagement in 4 or more early stimulation activities by other members of the household (15 years or older) in the past 3 days (according to the mother’s report).

The main predictor was the number of days the child was left with another adult or family member in the past week (none, 1 day, 2 days, 3 days or more). We believe that a child aged 0–5 years being left with another adult family member for at least 3 days or more would warrant closer attention as that is nearly half of the time during the week potentially being spent with non-primary caregivers in the household.

### Covariates

Several covariates were examined in the analysis models. Caregiver’s age was included because it theoretically correlates with parity and experience of caregiving which affect caregivers’ level of stimulation with the child. Child’s age was included because of prior studies that indicate family members’ shifting caregiving roles depending on the child’s age and stage of development [47,48]. Total number of females over 5 years old in the household was included to proxy available support for caregiving and household chores by other female adults or siblings in the household. Polygamous unions are also not uncommon in both Kenya [63] and Uganda [64]. Total number of children under 5 years old in the household was included as greater numbers of children in the household could theoretically indicate less caregiver attention and resources spent on the child. Sex of the child was included to control for any potential gender-based differences in caregivers’ early stimulation. There is prior evidence in Kenya that mothers engaged in greater early stimulation with female children compared to male children [65].

Experience of financial strain and caregivers’ stress over family responsibilities (never/rarely, sometimes, or always/quite frequently) were included because they could proxy caregivers’ availability and other household members’ need to care for the children. Financial strain could be driving women to participate in the labor force. In 2018, labor force participation rate of women in Sub-Saharan Africa was as high as 76% [66]. A large portion of these women engaged in unremunerated agricultural work on family farms [66]. There is also a growing trend for women to engage in income-generating activities outside of the family farm and domestic care work as well [66]. In addition, a UN Women’s survey report from Rwanda found that women in rural areas spent less than 1.5 hours a day caring for and teaching their children while fathers’ participation in caregiving and teaching children was about 42 minutes a day [67]. The total number of hours spent in unpaid domestic care work (i.e., household chores) by women amounted to about 7 hours a day [67]. Hence, the greater the financial strain and stress over family responsibilities, the more likely it may be that caregiving responsibility is shared amongst the members of the household.

Caregiver’s education was included because it may affect employment and subsequently, availability during the week to care for the child. It was coded as none/primary education or secondary or higher education. Marital status was included because living together with a partner could affect the engagement of fathers and others in the household. Marital status was coded as not married, divorced, separated, widowed or married or living with a partner. Receipt of information on child development and caregiver wellbeing was included because greater knowledge on the importance of early stimulation may affect the degree of their engagement. Ownership of children’s books and various types of toys were included because they are tools that enable mothers and other members of the household to stimulate and play with the child. Prior evidence in Kenya also found that presence of children’s books and toys were significantly associated with maternal early stimulation practices [65]. The effects of the covariates however are not presented as the focus of the study is to examine the effects of the main predictor.

### Analysis

Descriptive and multivariable analyses were performed with Stata SE 18.5 (Stata Corp, College Station, TX). The number of cases, means and frequencies were reported in descriptive Tables 1 and 2. For multivariable analyses, logistic regression was used to estimate the effects for binary outcomes with the aforementioned main predictor and covariates included in the models. For the outcome of paternal early stimulation, marital status was excluded from the model because of collinearity. Instead of reporting odds ratios, we calculated average marginal effects where each category of the main predictor is compared to the referent category for a percentage point difference in the probability of the outcome. Compared to odds ratios, average marginal effects have a more intuitive interpretation and are less sensitive to model specification changes [68]. Standard errors accounted for clustering at the community level and missing data techniques were not used as only about 3% of the analysis-samples were missing for both Kenya and Uganda. The reasons for missing data are unknown.

**Table 1:** Descriptive background information: Kenya and Uganda.

		Kenya		Uganda	
		N	%	N	%
**Background**					
	Caregiver’s age	1271	30.3	540	31.4
	Child’s age	1281	2.3	546	2.7
	Total number of females over 5 years old in the household	1273	2.1	542	2.7
	Total number of children under 5 years old in the household	1279	1.4	546	1.9
		**N**	**%**	**N**	**%**
	Experiencing financial strain				
	No	518	41%	222	41%
	Yes	761	60%	319	59%
	Caregiver’s education				
	None/primary education	637	50%	397	73%
	Secondary or higher education	644	50%	149	27%
	Marital status				
	Not married/divorced/separated/widowed	204	16%	52	10%
	Married or living with a partner	1073	84%	493	90%
	Child’s sex				
	Male	630	49%	254	47%
	Female	651	51%	292	53%
**Early child development knowledge and tools**					
	Received information on child development				
	No	605	47%	212	39%
	Yes	674	53%	332	61%
	Ownership of children’s books				
	No	1024	80%	446	82%
	Yes	256	20%	96	18%
	Ownership of homemade toys				
	No	418	33%	143	26%
	Yes	863	67%	403	74%
	Ownership of manufactured toys				
	No	721	56%	419	77%
	Yes	560	44%	127	23%
	Ownership of household objects as toys				
	No	571	45%	331	61%
	Yes	710	55%	215	39%
	Ownership of things that play music				
	No	1105	86%	471	86%
	Yes	176	14%	75	14%
	Ownership of things for drawing and writing				
	No	1151	90%	505	92%
	Yes	130	10%	41	8%
**Caregiver stress, absence, and knowledge of wellbeing**					
	Caregiver stress over family responsibilities				
	Never/rarely	427	33%	202	37%
	Sometimes	670	53%	281	52%
	Always/quite frequently	179	14%	62	11%
	Left child with another adult/family member in the past week				
	None	384	30%	182	33%
	1 day	286	22%	111	20%
	2 days	214	17%	90	17%
	3 days or more	395	31%	161	30%
	Received information about parenting or caregiver wellbeing in the community				
	No	717	56%	240	44%
	Yes	563	44%	304	56%

The samples only include responses from biological, maternal primary caregivers with children 0–5 years old.

**Table 2:** Early stimulation activities by different members of the household in Kenya and Uganda.

		Kenya		Uganda	
		N	Mean	N	Mean
**Number of early stimulation activities**					
	Engagement by any household member	1281	2.8	546	2.0
	Maternal engagement	1281	2.0	546	1.4
	Paternal engagement	1281	0.6	546	0.5
	Engagement by other members (15 years or older) of the household	1281	1.4	546	0.8
		**N**	**%**	**N**	**%**
**Engagement in 4 or more early stimulation activities**					
	Engagement by any household member				
	Less than 4	853	67%	456	84%
	4 or more	428	33%	90	16%
	Maternal engagement				
	Less than 4	1049	82%	477	87%
	4 or more	232	18%	69	13%
	Paternal engagement				
	Less than 4	1232	96%	522	96%
	4 or more	49	4%	24	4%
	Engagement by other members (15 years or older) of the household				
	Less than 4	1091	85%	515	94%
	4 or more	190	15%	31	6%

The samples only include responses from biological, maternal primary caregivers with children 0–5 years old.

### Research ethics

This study was exempt from IRB review (Category 4 Exemption) for analysis of secondary data from the Sterling IRB (#11523-ETKim). All participants of the original data collection provided written informed consent.

## Results

### Participant background

The average age of biological, maternal primary caregivers was around 30 years old in Kenya and 31 years old in Uganda (Table 1). The average age of the child was between 2 and 3 years old. There were also on average between 2 and 3 females over 5 years old and between 1 and 2 children under 5 years old in the household.

Over half of the caregivers reported experiencing financial strain (Kenya: 60%; Uganda: 59%). Half of the caregivers in Kenya had secondary education or higher (50%) while in Uganda, only about a quarter of the caregivers had secondary education or higher (27%). Most caregivers were married or living together with a partner (Kenya: 84%; Uganda: 90%). There were slightly more female children than there were male children in both samples (Kenya: 51%; Uganda: 53%).

### ECD knowledge and tools

A little over half of the caregivers in Kenya received information on child development (53%) and around 61% of Ugandan caregivers received such information (Table 1). There was little ownership of children’s books at home with 20% owning at least one children’s book in Kenya and 18% reporting ownership in Uganda. Homemade toys were the most abundant types of toys available at home (Kenya: 67%; Uganda: 74%), followed by household objects as toys (Kenya: 55%; Uganda: 39%). Ownership of manufactured toys was much less in comparison (Kenya: 44%; Uganda: 23%). Things that play music and materials used to draw and write were the least available at home with around 14% or less ownership.

### Caregiver stress, absence, and knowledge of wellbeing

Over 60% of caregivers reported that they had stress over family responsibilities sometimes, quite frequently or always (Kenya: 67%; Uganda: 63%) (Table 1). About a third of the caregivers also reported that they left their children with another adult or family member for 3 days or more in the past week (Kenya: 31%; Uganda: 30%). Forty-four percent of Kenyan caregivers and 56% of Ugandan caregivers reported having received information about parenting or caregiver wellbeing in the community.

### Early stimulation activities by different members of the household

Out of the 6 early stimulation activities assessed (reading books or looking at picture books with the child, telling stories to the child, singing songs to or with the child, taking the child outside the home, playing with the child, naming, counting or drawing things for or with the child), Kenyan mothers engaged in 2 activities on average (Table 2). Ugandan mothers engaged in about 1.4 activities. Other household members (15 years or older) engaged in about 1.4 activities in Kenya and close to 1 activity in Uganda. Fathers engaged in less than 1 activities on average (Kenya: 0.6; Uganda: 0.5).

In addition, about 18% of Kenyan mothers and 13% of Ugandan mothers engaged in at least four early stimulation activities. Paternal engagement in at least four early stimulation activities was much lower at 4% in Kenya and in Uganda. Engagement by other household members (15 years or older) was higher at 15% for Kenya and 6% for Uganda.

In summary, total engagement at the household level was on average 3 activities for Kenya and about 2 activities for Uganda. Engagement in 4 or more activities at the household level was around 33% in Kenya whereas in Uganda, it was around 16%.

### The effect of maternal absence on early stimulation activities

In Kenya, mothers leaving their child with another adult or family member for any number of days in the past week was not significantly associated with their engagement or fathers’ engagement in 4 or more early stimulation activities (Table 3). When mothers left their child for 3 days or more with another adult or family member, the likelihood of others’ engagement in 4 or more early stimulation activities significantly increased [Effect: 0.064; 95% CI: 0.033, 0.095].

**Table 3:** The effect of maternal absence on early stimulation activities by mothers, fathers, and others in the past 3 days.

	4 or more early stimulation activities by mother		4 or more early stimulation activities by father		4 or more early stimulation activities by others	
	Kenya	Uganda	Kenya	Uganda	Kenya	Uganda
Maternal absence^1^						
None	–	–	–	–	–	–
1 day	-0.017 [-0.06, 0.026]	-0.031 [-0.082, 0.021]	-0.025 [-0.056, 0.007]	-0.001 [-0.028, 0.027]	0.013 [-0.029, 0.054]	-0.005 [-0.016, 0.006]
2 days	0.021 [-0.035, 0.076]	0.061** [0.016, 0.107]	-0.004 [-0.064, 0.055]	0.052 [-0.012, 0.115]	0.025 [-0.025, 0.075]	0.018 [-0.031, 0.067]
3 days or more	0.038 [-0.02, 0.095]	0.078 [-0.003, 0.159]	0.024 [-0.019, 0.068]	0.058*** [0.026, 0.090]	0.064*** [0.033, 0.095]	0.013 [-0.026, 0.052]
**Observations** ^**2**^	1246	529	1246	530	1246	529

Dashes (-) represent referent categories.

95% confidence intervals in brackets.

* *p* < 0.05, ^**^ *p* < 0.01, ^***^ *p* < 0.001.

^1^Number of days biological, maternal primary caregivers left child with another adult/family member in the past week.

^2^The samples only include responses from biological, maternal primary caregivers with children 0–5 years old. All logistic regression models included covariates aforementioned in the Methods section.

In Uganda, mothers leaving their child with another adult or family member for 2 days in the past week was significantly associated with engagement in 4 or more early stimulation activities by themselves [Effect: 0.061; 95% CI: 0.016, 0.107]. Maternal absence for 3 days or more was significantly associated with fathers’ engagement in 4 or more early stimulation activities [Effect: 0.058; 95% CI: 0.026, 0.090]. Others’ engagement in 4 or more early stimulation activities was not significantly affected by maternal absence in Uganda.

## Discussion

Our findings contribute important evidence that young children in Kenya and Uganda spend a considerable amount of time with family members that may not necessarily be the identified primary caregiver (i.e., the person who primarily takes care of the child and is responsible for meeting most of the child’s needs). We specifically found that about a third of the children were left with another adult or family member for at least 3 days in the past week. Although the exact reasons for this in the study sample are unknown, we can speculate that they may have to do with employment or household chores as 60% of women reported financial strain and about two-thirds of women reported having stress over family responsibilities sometimes, quite frequently, or always.

The labor force participation rate of women in Sub-Saharan Africa was as high as 76% in 2018 [66]. A large portion of these women engage in unremunerated agricultural work on family farms and perform domestic care work [66]. A UN Women’s survey report from Rwanda also found that women in rural areas spent less than 1.5 hours a day caring for and teaching their children while fathers’ participation in caregiving and teaching children was about 42 minutes a day [67]. The total number of hours spent in unpaid domestic care work (i.e., household chores) by women amounted to about 7 hours a day [67]. Furthermore, there is a growing trend for women to engage in income-generating activities outside of the family farm and domestic care work as well [66]. This means there may be little time available for maternal caregivers to interact with their children during the day and other family members and relatives may be asked to step in to fill the gap [48].

Our findings have significant programmatic implications as maternal caregivers have often been prioritized and targeted for participation in ECD parenting programs [3]. While mothers are usually identified or may self-identify as the primary caregivers, the contribution of other family members such as fathers, grandparents, aunts, and older siblings cannot be overlooked in programming. This is especially true since time spent together with other family members did not always translate to an increase of their engagement in early stimulation. In fact, longer maternal absence in Uganda was significantly associated with greater paternal stimulation but not associated with stimulation by other family members. In contrast, longer maternal absence in Kenya was significantly associated with greater stimulation by other family members but not associated with paternal stimulation. We speculate that such differences may be potentially attributable to community social norms and household dynamics, especially considering the average household size, number of children, and presence of co-wives but more qualitative and participatory research is needed to elucidate the reasons for this finding. In any case, not enough children received the recommended dosage of 4 or more early stimulation activities at the household level which suggests a need for greater engagement overall.

Past qualitative studies in Burkina Faso and Malawi further corroborate our evidence and found that different members of the household took on the role of caregiver depending on the child’s age and stage of development [47,48]. Participants of the focus group sessions agreed that after infancy, the grandmother, co-wife, older sibling, or the father will spend more time with the child away from the mother [47,48]. However, activities with children were mostly instructive in nature rather than being responsive and stimulating [47].

Hence, our study findings as well as prior supporting evidence suggest that ECD interventions need to consider designing program content that is relevant for caregiving environments where multiple caregivers are involved. Incorporating programmatic elements specifically enhancing the quality of collective engagement by involved caregivers is therefore key and can potentially bring long-term benefits at different stages of children’s development. A low-cost yet pragmatic strategy to complement existing ECD program models may be to restructure at least some of the group sessions or home visits to include a brief didactic session with other family members (including fathers) on responsive and stimulating interactions. Community mobilization activities, digital delivery of ECD content and using mass media may also be feasible options [47]. For example, the Thrive by Five program is an app-based intervention which educates parents and caregivers on the “why” and the “how” of responsive caregiving based on a collectivist approach where the involvement of multiple caregivers is both acknowledged and encouraged [54,58].

Ownership of children’s books and toys at home also provides an important foundation for early stimulation [2]. Most surveyed households in Kenya and Uganda had some type of toys or play objects at home but ownership of children’s books was low on average. In low-resource settings, the most practical option has been to instruct caregivers on how to make basic age-appropriate toys using low-cost, locally available materials [69]. On the contrary, continually supplying high quality, age-appropriate, and culturally relevant children’s books in the local language may pose a greater challenge as programs tend to be short in duration and only target a specified age group whereby older cohorts age out and are replaced by newer and younger cohorts. Coupled with a programmatic solution, further investments in social advocacy and policy change may be necessary for a more sustainable solution.

## Limitations

A major limitation of this study is that it lacks contextual detail about unique circumstances in which the primary caregivers have to leave their children in the care of other adults for extended periods of time during the week. There is also a lack of understanding about how non-primary caregivers in the household perceive their roles and contributions in early stimulation and development. Such information would ideally be ascertained through in-depth qualitative interviews or focus groups. In addition, the current study only analyzed the responses of the biological, maternal primary caregivers and did not have a large enough sample size to conduct the same analyses when the primary caregiver was non-biological or was the child’s father or the grandmother. In cases where the primary caregiver is not the mother, the contribution of other family members may also take on a different meaning and significance, especially in the context of prevailing cultural and social norms. qualitative studies would be needed for understanding the fuller underlying context in such cases.

Nonetheless, our findings lend support to an old proverb that “it takes a village to raise a child.” Hence, we encourage program practitioners, policymakers, and development partners to consider adopting a holistic and contextualized strategy where all involved caregivers (including extended family members) are intentionally included in programs rather than just the identified primary caregivers and their partners alone.

## Conclusion

Nearly a third of the children aged 0–5 years in Kenya and Uganda were entrusted in the care of non-primary caregivers for at least 3 days or more during the week. Yet, the primary caregivers’ absence was not consistently associated with greater engagement in early stimulation by fathers and other family members. Our findings call for ECD parenting programs to consider adopting a holistic and contextualized approach where all involved caregivers are intentionally targeted rather than focusing just on the identified primary caregiver alone.

## Supporting information

S1 File Kenya data. (XLSX)

S2 File Uganda data. (XLSX)

S3 File Variable dictionary. (DOCX)

S4 File Analysis commands in Stata. (TXT)
