# Supplementary material for: The contribution of non-primary caregivers in early stimulation in Kenya and Uganda: Implications for ECD parenting programs in low- and middle-income countries
Source: PLoS One. 2025 May 23;20(5):e0323830. doi: 10.1371/journal.pone.0323830 (PMC12101776; doi:10.1371/journal.pone.0323830)
Supplement: S3 File — (DOCX) [file pone.0323830.s003.docx]

**Variable dictionary**

| **Variable name** | **Variable description** | **Response categories** |
| --- | --- | --- |
| mstim_read | Mother read books or looked at picture books with the child in the past 3 days | 1 = Yes  0 = No |
| mstim_stories | Mother told stories to the child in the past 3 days | 1 = Yes  0 = No |
| mstim_songs | Mother sang songs to or with the child (including lullabies) in the past 3 days | 1 = Yes  0 = No |
| mstim_outside | Mother took the child outside the home in the past 3 days | 1 = Yes  0 = No |
| mstim_play | Mother played with the child in the past 3 days | 1 = Yes  0 = No |
| mstim_ncd | Mother named, counted or drew things for or with the child in the past 3 days | 1 = Yes  0 = No |
| mstim_sum | A sum of the 6 early stimulation practice items by the mother (mstim_read, mstim_stories, mstim_songs, mstim_outside, mstim_play, mstim_ncd) | Continuous  (range of 0 to 6) |
| mstim_binary | Dichotomized version of the sum score for early stimulation practice by the mother | 1 = Four or more early stimulation practices by the mother  0 = Less than four early stimulation practices by the mother |
| fstim_read | Father read books or looked at picture books with the child in the past 3 days | 1 = Yes  0 = No |
| fstim_stories | Father told stories to the child in the past 3 days | 1 = Yes  0 = No |
| fstim_songs | Father sang songs to or with the child (including lullabies) in the past 3 days | 1 = Yes  0 = No |
| fstim_outside | Father took the child outside the home in the past 3 days | 1 = Yes  0 = No |
| fstim_play | Father played with the child in the past 3 days | 1 = Yes  0 = No |
| fstim_ncd | Father named, counted or drew things for or with the child in the past 3 days | 1 = Yes  0 = No |
| fstim_sum | A sum of the 6 early stimulation practice items by the father (fstim_read, fstim_stories, fstim_songs, fstim_outside, fstim_play, fstim_ncd) | Continuous  (range of 0 to 6) |
| fstim_binary | Dichotomized version of the sum score for early stimulation practice by the father | 1 = Four or more early stimulation practices by the father  0 = Less than four early stimulation practices by the father |
| ostim_read | Other household member read books or looked at picture books with the child in the past 3 days | 1 = Yes  0 = No |
| ostim_stories | Other household member told stories to the child in the past 3 days | 1 = Yes  0 = No |
| ostim_songs | Other household member sang songs to or with the child (including lullabies) in the past 3 days | 1 = Yes  0 = No |
| ostim_outside | Other household member took the child outside the home in the past 3 days | 1 = Yes  0 = No |
| ostim_play | Other household member played with the child in the past 3 days | 1 = Yes  0 = No |
| ostim_ncd | Other household member named, counted or drew things for or with the child in the past 3 days | 1 = Yes  0 = No |
| ostim_sum | A sum of the 6 early stimulation practice items by other household member (ostim_read, ostim_stories, ostim_songs, ostim_outside, ostim_play, ostim_ncd) | Continuous  (range of 0 to 6) |
| ostim_binary | Dichotomized version of the sum score for early stimulation practice by other household member | 1 = Four or more early stimulation practices by other household member  0 = Less than four early stimulation practices by other household member |
| anystim_read | Any household member read books or looked at picture books with the child in the past 3 days | 1 = Yes  0 = No |
| anystim_stories | Any household member told stories to the child in the past 3 days | 1 = Yes  0 = No |
| anystim_songs | Any household member sang songs to or with the child (including lullabies) in the past 3 days | 1 = Yes  0 = No |
| anystim_outside | Any household member took the child outside the home in the past 3 days | 1 = Yes  0 = No |
| anystim_play | Any household member played with the child in the past 3 days | 1 = Yes  0 = No |
| anystim_ncd | Any household member named, counted or drew things for or with the child in the past 3 days | 1 = Yes  0 = No |
| anystim_sum | A sum of the 6 early stimulation practice items by any household member (anystim_read, anystim_stories, anystim_songs, anystim_outside, anystim_play, anystim_ncd) | Continuous  (range of 0 to 6) |
| anystim_binary | Dichotomized version of the sum score for early stimulation practice by any household member | 1 = Four or more early stimulation practices by any household member  0 = Less than four early stimulation practices by any household member |
| left_wadult | Left child with another adult/family member in the past week | 1 = None  2 = 1 day  3 = 2 days  4 = 3 days or more |
| stress_over_family | Caregiver stress over family responsibilities | 1 = Never/rarely  2 = Sometimes  3 = Always/quite frequently |
| cg_finstrug | Experiencing financial strain | 1 = Yes  0 = No |
| clusterid | Community clusters (de-identified) | Used for clustered standard errors |
| femalesexofchild | Child’s sex | 1 = Female  0 = Male |
| married | Marital status | 1 = Married or living with a partner  0 = Not married/divorced/  separated/widowed |
| edu2 | Caregiver’s education | 1 = Secondary or higher education  0 = None/primary education |
| num_adultyouth_females | Total number of females over 5 years old in the household | Continuous  (range of 0 to 9) |
| tot_num_under5 | Total number of children under 5 years old in the household | Continuous  (range of 0 to 10) |
| ecdknow | Received information on child development | 1 = Yes  0 = No |
| childbooks | Ownership of children’s books | 1 = Yes  0 = No |
| homemadetoys | Ownership of homemade toys | 1 = Yes  0 = No |
| manutoys | Ownership of manufactured toys | 1 = Yes  0 = No |
| HHobjecttoys | Ownership of household objects as toys | 1 = Yes  0 = No |
| musicthings | Ownership of things that play music | 1 = Yes  0 = No |
| drawwritetools | Ownership of things for drawing and writing | 1 = Yes  0 = No |
| cg_wellbeing_info | Received information about parenting or caregiver wellbeing in the community | 1 = Yes  0 = No |
| cg_age | Caregiver’s age in years | Continuous |
| childage | Child’s age in years | Continuous |
